# Objective allergy markers and risk of cancer mortality and hospitalization in a large population-based cohort

**DOI:** 10.1007/s10552-014-0489-9

**Published:** 2014-11-12

**Authors:** Niloofar Taghizadeh, Judith M. Vonk, Jeannette J. Hospers, Dirkje S. Postma, Elisabeth G. E. de Vries, Jan P. Schouten, H. Marike Boezen

**Affiliations:** 1 Department of Epidemiology, University Medical Center Groningen, University of Groningen, Groningen, The Netherlands; 2 GRIAC Research Institute, University Medical Center Groningen, University of Groningen, Groningen, The Netherlands; 3 Department of Pulmonology, University Medical Center Groningen, University of Groningen, Groningen, The Netherlands; 4 Department of Medical Oncology, University Medical Center Groningen, University of Groningen, Groningen, The Netherlands

**Keywords:** Cancer mortality, Cancer hospitalization, Eosinophils, IgE, Skin test positivity

## Abstract

**Purpose:**

There are indications that a history of allergy may offer some protection against cancer. We studied the relation of three objectively determined allergy markers with cancer mortality and hospitalization risk.

**Methods:**

Associations between three allergy markers (number of peripheral blood eosinophil counts, skin test positivity, and serum total IgE) with mortality and hospitalization from any type and four common types of cancer (lung, colorectal, prostate, and breast cancer) were assessed in the Vlagtwedde–Vlaardingen cohort (1965–1990), with follow-up of mortality until 31 December 2008. Hospitalization data were available since 1 January 1995.

**Results:**

There were no significant associations between objective allergy markers and cancer mortality or hospitalization. We found several associations in specific subgroups. A higher number of eosinophils was associated with a decreased risk of colorectal cancer mortality in ever smokers HR (95 % CI) = 0.61 (0.45–0.83) and in males 0.59 (0.42–0.83); however, no overall association was observed 0.84 (0.64–1.09). Skin test positivity was associated with a decreased risk of any cancer mortality only among females 0.59 (0.38–0.91) and showed no overall association 0.83 (0.67–1.04). Serum total IgE levels were associated with an increased risk of lung cancer mortality among females 4.64 (1.04–20.70), but with a decreased risk of cancer hospitalization in ever smokers 0.77 (0.61–0.97) and males 0.72 (0.55–0.93); however, no overall associations were observed [mortality 0.99 (0.79–1.25), and hospitalization 0.86 (0.71–1.04)].

**Conclusions:**

We found no associations between objective allergy markers and cancer in the total population. However, skin test positivity and a high number of eosinophils were associated with a reduced risk to die of cancer in specific subgroups. Hence, it seems important to study specific subgroups defined by gender and smoking habits in order to identify allergy markers of predictive value for cancer mortality.

**Electronic supplementary material:**

The online version of this article (doi:10.1007/s10552-014-0489-9) contains supplementary material, which is available to authorized users.

## Introduction

There are indications of an inverse association between a history of allergy and cancer [1–3], suggesting that allergies may offer some protection against cancer in general. Subjects with allergy have a hyperactive immune system. The fact that the immune system can continually recognize and remove malignant cells might explain such a protective effect of allergy on cancer development [1]. Although an appealing theory, the strength of the evidence in favor of this so-called immune surveillance theory is limited. Studies on the association between allergy and cancer show inconsistent results, mainly because the association between allergy and cancer is complex and is based on both different types of cancer and different definitions of allergy [3].

Thus, some studies have reported a negative association between allergy and cancer, supporting the immune surveillance theory [1–3], while others show a positive [5, 6] or no general association [4]. A theory that is often used to explain increased risk of specific cancers associated with allergy is the ‘antigenic stimulation theory.’ It suggests that inflammatory conditions associated with allergic diseases may induce the oxidative damage, resulting in tumor suppressor gene mutations in proteins involved in DNA repair or apoptotic control, thus may increase the development of cancer [7, 8]. Besides the above-mentioned mechanism, there is an emerging evidence for an important role of T-helper 2 (TH2) immune skewing in the association between allergy and cancer [8].

Recent reviews showed that the association between allergies and cancer is organ or site specific [6]. Several studies indicated that the presence of allergy markers was, for example, associated with a decreased risk of colorectal cancer, pancreatic cancer, and larynx cancer, but with increased risks of lymphoma, prostate cancer, and myeloma, and there are inconsistent results for breast cancer and lung cancer [6, 8, 9].

However, many unanswered questions remain about the inconsistently reported associations between allergy and cancer risk. Few studies have reported whether the associations between allergy and cancer risk vary according to gender or smoking habits [8]. For instance, Hsiao et al. [10] found an inverse association between allergies and head and neck cancer, particularly among males and smokers. However, no clear explanation for the gender difference has been proposed. Thus, future studies are needed to clarify the role of smoking and gender in the association between allergy and different cancer types.

We studied in a general population sample in two Dutch communities (Vlagtwedde–Vlaardingen) whether allergy is associated with cancer mortality and hospitalization (as proxies for cancer incidence) after adjustment for potential confounders. We also assessed the possible effect modification of gender and smoking on the association between allergy and cancer since previous studies suggested these might have differential effects [2, 11].

## Methods

### Ethics statement

The Committee on Human Subjects in Research of the University of Groningen reviewed the study and affirmed the safety of the protocol and study design and specifically approved this study. All participants gave their written informed consent.

### Study population

We studied objective allergy markers, cancer mortality and hospitalization using the Vlagtwedde–Vlaardingen cohort study. The Vlagtwedde–Vlaardingen study was set up as a general population-based cohort study on the epidemiology of pulmonary diseases in exclusively Caucasian individuals of Dutch descent [12, 13]. This study started in 1965 and participants had medical examinations every 3 years until the last survey in 1989/1990. In Vlaardingen, only participants who were included at baseline (1965 or 1969) were approached for follow-up, whereas in Vlagtwedde new subjects aged between 20 and 65 were invited to participate at every survey. We updated the vital status of all participants on 31 December 2008 and evaluated five main cancer mortality outcomes, i.e., mortality from all types of cancer, lung, colorectal, prostate, and breast, either as primary or secondary cause of death. The causes of death were coded according to the International Classification of Diseases (ICD). Hospitalization data since 1 January 1995 were obtained using probabilistic matching based on date of birth, gender, and postal code. The probabilistic matching method was used because of privacy regulations. A match was defined if date of birth, gender, and postal code in our source-file (the Vlagtwedde–Vlaardingen cohort data) were exactly equal to those in the hospital admission registry file. Diagnosis at discharge was used to identify the reason for the hospitalization. The endpoints used for the current study were having at least one hospitalization due to any cancer or due to a specific type of cancer (i.e., lung, colorectal, prostate, and breast cancer). Subjects who were lost to follow-up or died within 2 years after the start of the hospitalization data (in 1995) and who were not hospitalized during these years were excluded from these analyses. However, subjects with a shorter registration period but with a hospital admission for cancer in this period are included in the group ‘at least one hospitalization due to any cancer.’

### Population characteristics

We collected data on age, gender, and smoking habits using the Dutch version of the British Medical Research Council questionnaire [12, 13]. We used the data of a subject’s first available survey. We defined smoking habits as follows: Never smoker and ever (i.e., ex and current) smoker (including pipe/cigar smokers).

The body mass index (BMI) was calculated as weight in kilograms divided by the square of the height in meters (kg/m^2^).

### Allergy

Peripheral blood eosinophil counts were assessed in a 1:11 dilution of peripheral blood with a Bürker counting chamber [12, 13].

Skin prick tests were performed at the first available survey. Four common aeroallergens (house dust, mixed pollen, epidermal products, and mixed molds) were applied intracutaneously to the forearm (Diephuis, Groningen, the Netherlands) [14]. Wheal diameters for each allergen were measured to the nearest half millimeter and coded on a six-point scale (0 = 0–5.0 mm, 1 ≥ 5.0–7.5 mm, 2 ≥ 7.5–10.0 mm, 3 ≥ 10.0–12.5 mm, 4 ≥ 12.5 mm, 5 ≥ 15.0 mm). Scores for the four allergens were added to a skin test sum score (minimum 0, maximum 20). Skin test positivity was defined as a skin test sum score ≥3 [15].

Serum total Immunoglobulin E (IgE) was determined at only one survey, i.e., the final survey, with the CAP system (Pharmacia, Woerden, the Netherlands) and expressed in kU/L [15].

### Cancer mortality and hospitalization

Cancer was classified according to the ICD-coding system: Any type of cancer (ICD 7: 140–239 and 294; ICD 8: 140–239; ICD 9: 140–239 and 288; ICD10: C00-C97, D00-D48), lung cancer (cancer of trachea, bronchus, and lung) (ICD 7: 162, 163; ICD 8: 162, 163; ICD 9: 162, 163, 165; ICD10: C33, C34, C38, C39), cancer of colon and rectum (further referred to as colorectal cancer) (ICD 7: 153, 154; ICD 8: 153, 154; ICD 9: 153, 154; ICD 10 C18-C21), breast cancer (ICD 7: 170; ICD 8: 174; ICD 9:174, 175 and ICD10: C50), and prostate cancer (ICD 7: 177; ICD 8: 185; ICD 9: 185 and ICD 10: C61).

### Statistical analyses

Descriptive analyses of the subject characteristics and the mortality and hospitalization data were performed. Differences between groups were tested with independent samples *t* test and Chi-square test for continuous and categorical variables, respectively. Blood eosinophil counts and serum total IgE were log-transformed to obtain normality of the distribution. Multivariate Cox regression (for mortality) and logistic regression (for hospitalization) with adjustment for age, gender, Forced Expiratory Volume in 1 s (FEV_1_) as % of predicted, BMI (all at the first survey), and place of residence were used to estimate the effect of the allergy markers on the cancer outcomes. To determine whether the association of the three allergy markers was different for males and females, or for ever and never smokers, stratified analyses were performed, and interactions between allergy markers and gender, or smoking were tested. In the Cox regression analyses, censoring took place when the subjects were still alive, were lost to follow-up, or died of causes other than cancer or the specific cancer under study [16]. Time was defined from the first available survey until cancer mortality or until censoring in the analyses of eosinophils and skin test positivity. Similarly, in the analyses of IgE, time was defined from the only available survey, i.e., the final survey, until cancer mortality or until censoring. Finally, to investigate the robustness of our results, we conducted several sensitivity analyses. All analyses were performed at Statistics Netherlands (The Hague, the Netherlands). *p* values <0.05 (two sided) were considered to be statistically significant.

## Results

### Mortality

**Fig. 1:**
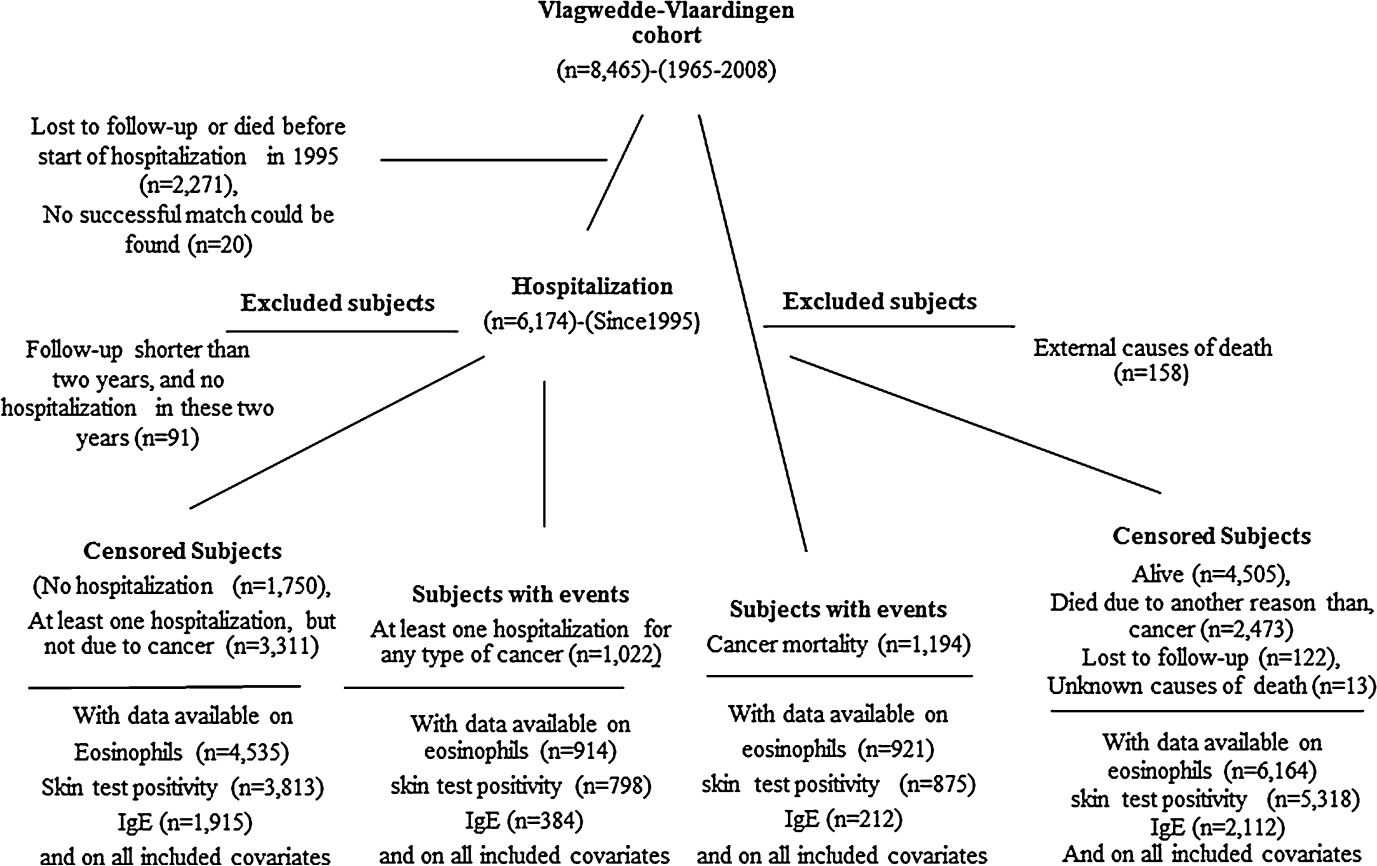
Flow diagram showing the Vlagtwedde–Vlaardingen study design

Among all 8,465 subjects, 4,505 (53.2 %) were alive, 1,194 (14.1 %) died due to cancer, 2,473 (29.2 %) died due to another reason than cancer, 158 subjects (1.9 %) died due to external causes such as an accident, suicide or homicide, in 13 (0.1 %) subjects the cause of death could not be determined, and 122 (1.5 %) subjects were lost to follow-up (Table 1). Of those subjects who died due to cancer, most died of lung cancer (*n* = 275, 23.0 %), followed by colorectal cancer (*n* = 134, 11.2 %), prostate cancer (*n* = 83, 7.0 %), and breast cancer (*n* = 117, 9.9 %) (Fig. 1).

**Table 1:** Characteristics at the first survey of 8,465 subjects according to vital status in 2008

Characteristic	Alive (*n* = 4505) (A)	Died due to cancer (*n* = 1,194) (DC)	Died, but not due to cancer (*n* = 2,473) (DNC)	Died due to external causes (*n* = 158)	Lost to follow-up (*n* = 122)	*p* value^e^ DC versus A	*p* value^e^ DC versus DNC
All subjects (%)^a^	53.2	14.1	29.3	1.9	1.5		
Male (%)	48.8	58.3	54.8	63.9	57.4	0.00	0.04
Age (years) [mean (SD)]	30.2 (10.2)	45.9 (11.1)	50.1 (9.6)	43.6 (13.7)	33.2 (13.3)	0.00	0.00
Smoking (%)							
Never smoker	38.0	33.2	39.8	35.9	38.8	0.00	0.00
Ever smoker	62.0	66.8	60.2	64.1	61.2		
FEV_1_ % of predicted^b^ [mean (SD)]	90.4 (12.4)	85.2 (14.6)	82.9 (16.7)	87.2 (13.2)	89.6 (12.1)	0.00	0.00
BMI (kg/m^2^) (%)							
<25	60.9	39.4	32.4	50.8	64.7		
25–30	31.8	44.7	49.5	37.9	29.3	0.00	0.00
>30	7.3	15.9	18.0	11.3	6.0		
Eosinophil count (*11 cells/µl) (Ln) [mean (SD)]	2.3 (0.8)	2.4 (0.7)	2.5 (0.7)	2.3 (0.9)	2.4 (0.9)	0.00	0.52
Skin test positivity (%)	18.9	9.9	9.5	13.6	27.1	0.00	0.71
Serum total IgE (kU/L) (Log 10), mean (SD)^c^	1.4 (1.6)	1.4 (0.6)	1.5 (0.6)	1.5 (0.7)	1.3 (0.5)	0.91	0.30
Follow-up time for eosinophils in years, median (range)^d^	39.2 (19.2–44.2)	26.8 (0.5–43.2)	26.9 (0.2–43.2)	19.6 (0.2–42.8)	22.8 (0.0–41.0)	0.00	0.36
Follow-up time for skin test positivity in years, median (range)^d^	39.2 (36.2–44.2)	27.4 (0.5–43.2)	27.6 (0.2–43.2)	12.1 (0.2–42.8)	26.2 (0.0–41.0)	0.00	0.85
Follow-up time for serum total IgE in years, median (range)^d^	19.2 (18.2–19.2)	11.8 (0.6–19.2)	12.8 (0.4–19.2)	13.3 (2.0–18.7)	12.2 (1.1–18.7)	0.00	0.21
Place of residence (%)							
Vlagtwedde	66.7	63.8	69.5	72.8	55.7	0.09	0.00

^a^All subjects: *n* = 8,452; in 13 subjects, the cause of death could not be determined

^b^FEV_1_ % of predicted, percentage of predicted forced expiratory volume in 1 s

^c^IgE was measured at the last survey in 1989/1990

^d^The follow-up time for allergy markers were calculated as the difference between the age at the first available allergy markers measurement and the age at last known vital status

^e^ *p* value calculated by Chi square or *t* test

Details on the associations between allergy markers and baseline characteristics of the subjects in 1965/1967/1969 and vital status in 2008 are presented in the Online Resource Tables 1 and 2, respectively.

Among all 8,465 subjects, 7,085 (83.7 %) subjects had data available on peripheral blood eosinophil counts and on all included covariates, 6,193 (73.1 %) had data available on skin test positivity and on all included covariates, and for 2,324 (27.5 %) subjects, data on IgE and on all included covariates could be obtained (Online Resource Table 3).

Subjects who died due to cancer were more often men, older, had a higher BMI, were more often smokers, and had a lower FEV_1_ % predicted at the first survey than those who were alive. Subjects who died due to cancer had a higher level of peripheral blood eosinophils compared with subjects who were alive. Subjects who died due to cancer had less skin test positivity compared with subjects who were alive (*p* = 0.001). There were no significant differences in the level of serum total IgE between subjects who died due to cancer and those who were alive (Table 1).

### Hospitalization

Of the total number of 8,465 subjects, 6,174 subjects were successfully matched to the hospital admission file (in 20 subjects, a successful match could not be found, and 2,271 subjects were lost to follow-up or died before the start of registration of hospitalization in 1995). Of these 6,174 subjects, we excluded 91 subjects because they had a follow-up period shorter than 2 years and no hospital admission in these 2 years after the start of registration of hospitalization (Fig. 1). Among all 6,083 subjects with data on hospitalization, 1,022 (16.8 %) subjects were hospitalized for any type of cancer. Subjects, who had at least one hospitalization due to cancer were significantly older, were more often smokers, had a higher BMI, and had a lower FEV_1 _% predicted compared with subjects who had no hospitalization. Subjects with at least one hospitalization due to cancer were less often skin test positive compared with subjects who had no hospitalization (*p* = 0.02). Subjects with at least one hospitalization due to cancer were older compared with subjects who had at least one hospitalization but not due to cancer (*p* = 0.03). There were no significant differences in the level of eosinophils, and the level of serum total IgE, between subjects who had at least one hospitalization for any type of cancer and those who were not hospitalized at all or those who were hospitalized but not due to cancer (Table 2).

**Table 2:** Characteristics at first survey of subjects according to hospitalization status

Characteristics	No hospitalization (*n* = 1,750) (NA)	At least one hospitalization for any type of cancer (*n* = 1,022) (CA)	At least one hospitalization but not due to cancer (*n* = 3,311) (NCA)	*p* value^b^ CA versus NA	*p* value^b^ CA versus NCA
Age on 1 January 1995 (years) [mean (SD)]	54.2 (12.0)	61.7 (11.7)	60.5 (12.4)	0.00	0.00
Male (%)	46.9	51.3	48.8	0.03	0.16
Smoking (%)					
Never smoker	41.2	33.0	38.3	0.00	0.00
Ever smoker	58.8	67.0	61.7		
FEV_1_ % of predicted^a^ [mean (SD)]	90.2 (12.1)	88.2 (13.6)	89.4 (13.1)	0.00	0.01
BMI (kg/m^2^) (%)					
<25	67.5	46.5	51.1		
25–30	26.1	41.9	38.7	0.00	0.04
>30	6.4	11.6	10.2		
Eosinophil count (*11 cells/µl) (Ln) [mean (SD)]	2.4 (0.8)	2.4 (0.8)	2.4 (0.8)	0.74	0.75
Skin test positivity (%)	19.5	15.5	16.2	0.02	0.65
Serum total IgE (kU/L) (Log 10) at visit 1989/1990 [mean (SD)]	1.5 (0.6)	1.4 (0.6)	1.4 (0.6)	0.09	0.23
Place of residence (%)					
Vlagtwedde	63.0	65.6	69.0	0.17	0.04

Hospitalization registry data were available for 6,083 subjects (3,125 females and 2,925 males)

^a^FEV_1_ % of predicted, percentage of predicted forced expiratory volume in 1 s

^b^ *p* value calculated by Chi-square or *t* test

Among all 6,083 subjects with data on hospitalization, 5,449 (89.6 %) subjects had data available on peripheral blood eosinophil counts and on all included covariates, 4,611 (75.8 %) had data on skin test positivity and on all included covariates, and 2,299 (37.8 %) subjects had data on IgE and on all included covariates (Online Resource Table 3).

### Peripheral blood eosinophil counts

**Fig. 2:**
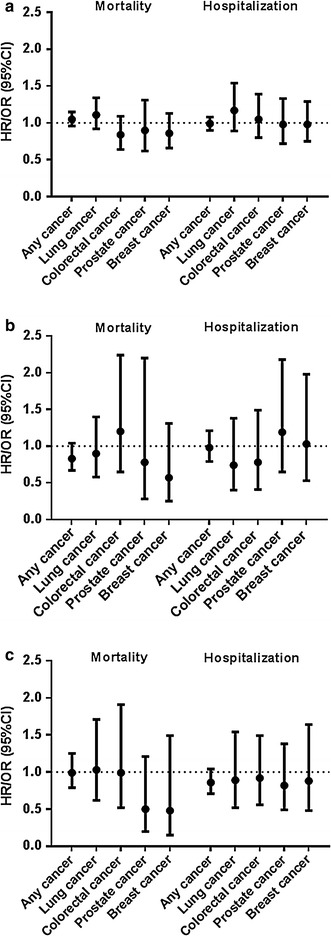
Hazard ratio of eosinophils (**a**), skin test positivity (**b**), and IgE (**c**) for mortality and Odds ratio for hospitalization from cancer

In the total population, we found no significant association between number of eosinophils and cancer mortality or cancer hospitalization (Table 3; Fig. 2a). A higher number of eosinophils was significantly associated with decreased risk of colorectal cancer mortality in ever smokers (Hazard ratio (HR) (95 % confidence interval (CI)) = 0.61 (0.45–0.83); see Table 4) and males (0.60 (0.42–0.83); Table 5) (Online Resource Figure 1 and 2). The interaction between the number of eosinophils and smoking and gender was significant. To assess whether this association is gender or smoking dependent, we investigated the interaction between eosinophils and smoking separately in males and females and the interaction between eosinophils and gender separately in never and ever smokers. We observed a significant interaction between eosinophils and ever smoking within males and a significant interaction between eosinophils and male gender within ever smokers (Online Resource Table 4). There were no significant interactions between number of eosinophils and gender or smoking in the analyses on hospitalization due to cancer (Online Resource Table 5).

**Table 3:** Hazard ratio of allergy markers for mortality, and odds ratios of allergy markers for hospitalization from any and specific type of cancer

	Any cancer	Lung cancer	Colorectal cancer	Prostate cancer	Breast cancer
Cancer mortality	HR (95 % CI)	HR (95 % CI)	HR (95 % CI)	HR (95 % CI)	HR (95 % CI)
Eosinophils (ln)	1.05 (0.96–1.15)	1.11 (0.92–1.34)	0.84 (0.64–1.09)	1.02 (0.71–1.46)	0.86 (0.66–1.13)
Skin test positivity	0.83 (0.67–1.04)	0.90 (0.58–1.40)	1.20 (0.65–2.24)	0.69 (0.24–1.94)	0.57 (0.25–1.31)
Total IgE (log10)	0.99 (0.79–1.25)	1.03 (0.62–1.71)	0.99 (0.52–1.91)	0.59 (0.26–1.36)	0.48 (0.15–1.49)
Cancer hospitalization	OR (95 % CI)	OR (95 % CI)	OR (95 % CI)	OR (95 % CI)	OR (95 % CI)
Eosinophils (ln)	0.99 (0.90–1.08)	1.17 (0.89–1.54)	1.05 (0.80–1.39)	0.98 (0.72–1.33)	0.98 (0.75–1.29)
Skin test positivity	0.98 (0.79–1.21)	0.74 (0.40–1.38)	0.78 (0.41–1.49)	1.19 (0.65–2.18)	1.03 (0.53–1.98)
Total IgE (log10)	0.86 (0.71–1.04)	0.89 (0.52–1.54)	0.92 (0.56–1.49)	0.82 (0.49–1.38)	0.88 (0.48–1.64)

**Table 4:** Interaction of eosinophils, skin test positivity, and IgE with smoking on mortality risk from any type of cancer, lung cancer, colorectal cancer, prostate cancer (only in males), and breast cancer (only in females)

	Any cancer HR (95 % CI)	*p* value	Lung cancer HR (95 % CI)	*p* value	Colorectal cancer HR (95 % CI)	*p* value	Prostate cancer HR (95 % CI)	*p* value	Breast cancer HR (95 % CI)	*p* value
*Eosinophils (ln)*										
Effect in never smokers	1.09 (0.92–1.28)	0.323	0.82 (0.37–1.82)	0.624	1.52 (0.96–2.40)	0.073	1.30 (0.47–3.55)	0.613	0.92 (0.66–1.28)	0.601
Effect in ever smokers	1.03 (0.93–1.15)	0.583	1.13 (0.93–1.37)	0.210	**0.61 (0.45–0.83)**	0.002	0.98 (0.67–1.44)	0.922	0.78 (0.50–1.20)	0.257
Interaction	0.95 (0.78–1.15)	0.597	1.38 (0.61–3.14)	0.442	**0.40 (0.23–0.70)**	0.001	0.76 (0.26–2.22)	0.611	0.85 (0.49–1.47)	0.552
*Skin test positivity*										
Effect in never smokers	0.81 (0.52–1.26)	0.355	1.12 (0.14–9.02)	0.913	1.26 (0.44–3.58)	0.571	1.12 (0.13–9.44)	0.920	0.61 (0.22–1.70)	0.349
Effect in ever smokers	0.84 (0.65–1.09)	0.184	0.89 (0.57–1.40)	0.624	1.18 (0.55–2.51)	0.670	0.61 (0.19–1.99)	0.414	0.49 (0.12–2.08)	0.335
Interaction	1.03 (0.62–1.71)	0.897	0.80 (0.10–6.67)	0.833	0.94 (0.26–3.38)	0.923	0.55 (0.05–6.18)	0.626	0.80 (0.14–4.66)	0.805
*Total IgE (log10)*										
Effect in never smokers	0.88 (0.51–1.51)	0.446	2.03 (0.21–19.97)	0.717	0.75 (0.16–3.58)	0.717	1.21 (0.00–5.41)	0.998	0.44 (0.12–1.57)	0.209
Effect in ever smokers	1.02 (0.79–1.31)	0.891	1.00 (0.59–1.68)	0.981	1.05 (0.52–2.15)	0.882	0.59 (0.26–1.36)	0.216	0.69 (0.06–7.67)	0.765
Interaction	1.16 (0.64–2.10)	0.632	0.49 (0.05–5.09)	0.696	1.41 (0.25–7.88)	0.696	0.49 (0.00–2.19)	0.994	1.58 (0.10–23.99)	0.744

Statistically significant results are shown in bold

**Table 5:** Interaction of eosinophils, skin test positivity, and IgE with gender on mortality risk from any type of cancer, lung cancer, and colorectal cancer

	Any cancer HR (95 % CI)	*p* value	Lung cancer HR (95 % CI)	*p* value	Colorectal cancer HR (95 % CI)	*p* value
*Eosinophils (ln)*						
Effect in females	1.04 (0.91–1.19)	0.551	1.35 (0.89–2.05)	**0.159**	1.25 (0.84–1.85)	0.272
Effect in males	1.05 (0.93–1.18)	0.419	1.06 (0.86–1.30)	**0.584**	**0.59 (0.42–0.83)**	0.003
Interaction	1.01 (0.84–1.21)	0.936	0.79 (0.49–1.25)	**0.307**	**0.48 (0.28–0.80)**	0.005
*Skin test positivity*						
Effect in females	**0.59 (0.38–0.91)**	0.016	0.79 (0.24–2.59)	**0.694**	0.74 (0.23–2.40)	0.613
Effect in males	0.97 (0.75–1.26)	0.821	0.92 (0.57–1.48)	**0.737**	1.55 (0.74–3.22)	0.244
Interaction	**1.65 (1.00-2.73)**	0.051	1.17 (0.33–4.21)	**0.809**	2.10 (0.53–8.30)	0.293
*Total IgE (log10)*						
Effect in females	1.18 (0.77–1.80)	0.475	**4.64 (1.04–20.70)**	**0.040**	0.68 (0.21–2.19)	0.506
Effect in males	0.95 (0.73–1.25)	0.659	0.85 (0.50–1.45)	**0.611**	1.22 (0.57–2.61)	0.638
Interaction	0.81 (0.49–1.33)	0.371	**0.18 (0.04–0.90)**	**0.031**	1.79 (0.44–7.23)	0.428

Statistically significant results are shown in bold

### Skin test positivity

Skin test positivity was not associated with cancer mortality or cancer hospitalization in the total population (Table 3; Fig. 2b). Within females, skin test positivity was associated with a decreased risk of mortality from any type of cancer (0.59 (0.38–0.91); Table 5), and the interaction between skin test positivity and gender on any type of cancer mortality was significant. There were no significant interactions between skin test positivity and gender on any type of cancer hospitalizations (Online Resource Table 6).

### Serum total IgE

Serum total IgE was not associated with cancer mortality or cancer hospitalization in the total population (Table 3; Fig. 2c). The association between total IgE and cancer mortality risk was not significantly different between ever and never smokers (Table 3). The association between serum total IgE and cancer mortality risk was significantly different between males and females, with a significantly increased risk of mortality from lung cancer among females (4.64 (1.04–20.70)) (Table 4). Higher levels of serum total IgE were associated with a lower chance of hospitalization for all types of cancer among males (0.76 (0.59–0.98) (Online Resource Table 6).

### Sensitivity analyses

A sensitivity analysis on cancer mortality, excluding the subjects who were lost to follow-up or died within 2 years of the visit with the assessment of the allergy marker [(eosinophils: total *n* = 44, cancer mortality *n* = 10), (skin test positivity: total *n* = 41, cancer mortality *n* = 7), and (total IgE: total *n* = 30, cancer mortality *n* = 12)], gave similar results as the main analysis (results not shown).

In addition, we performed separate analyses on cancer as primary cause of death and as secondary cause of death. The results of these analyses were comparable to the results of the presented analyses where we analyzed cancer as either the primary or secondary cause of death (results not shown). Since FEV_1_ could be on a causal path from allergy to cancer mortality, we performed a sensitivity analyses excluding FEV_1_ from our Cox regression model. These analyses gave the same results as our main analyses (results not shown).

The different inclusion strategy of Vlagtwedde and Vlaardingen may introduce bias. We, therefore, stratified our analyses by place of residence. In addition, we performed a meta-analysis with these two studies and demonstrated evidence of association between both datasets. The meta-analysis showed the same result as the original pooled analysis (results not shown).

Furthermore, additional adjustment for years of recruitment gave the same results as our main analyses (results not shown).

## Discussion

This is the first large cohort study that investigated three objective markers of allergy, mortality and hospitalization due to cancer in the general population. We found no association between allergy and the risk to die of cancer or hospitalization in the total population. However, in specific subgroups, we did find such associations: Higher numbers of eosinophils were associated with a reduced risk of colorectal cancer mortality among ever smokers and males. The effect of skin test positivity on the risk of mortality from all types of cancer was different for males and females, and we found a negative association among females. The effect of IgE on lung cancer mortality risk was different for males and females; we found a positive association among females. Higher levels of serum total IgE showed to be protective against all types of cancer hospitalizations among males and ever smokers.

The findings of the current study corroborate the findings of previous work in this field; especially those who found no general association between allergy and cancer, with the same definition of allergy as we used [17–20], or based on a self-reported history of allergy [21]. Results of previous studies were inconsistent. This type of discrepancy between results among studies is understandable, mainly because the association between allergies and cancer is complex and is based on both different types of cancer [5] and different definitions of allergy [8, 11]. Studies vary considerably in their definitions of allergy and allergy markers. For instance, very few studies distinguish between atopy [type-I allergy, IgE-mediated hypersensitivity] and allergy [immune hypersensitivity, regardless of the mechanism] [11].

Although immune surveillance and antigen stimulation are the most established hypotheses for explaining the association between allergy and cancer, the body of current worldwide literature provides limited support for these two hypotheses [19].

We found that high numbers of peripheral blood eosinophils are protective against colorectal cancer mortality only in males and ever smokers. In-depth analyses showed that a decreased risk of mortality from colorectal cancer is associated with a high number of eosinophils within males who were smokers (Online Resource Table 4). This observation may be explained by the fact that in our study population, smokers and males had a higher number of eosinophils compared with females and non-smokers, suggesting a threshold effect. This means that the protective effect of eosinophils on cancer only becomes apparent given a certain minimal level of eosinophils. However, an exploratory analysis in which we divided the eosinophil levels into equally spaced categories showed no evidence of this threshold effect (Online Resource Table 7).

Another explanation may be that although higher numbers of blood eosinophils are an important aspect of allergy, in smokers, eosinophil levels may be a better indicator of general inflammation rather than allergic inflammation. Earlier results in the Vlagtwedde–Vlaardingen cohort indeed showed within smokers the relationship between eosinophils and allergy is weaker than in non-smokers [22]. Since general inflammation is a risk factor for many other diseases (such as cardiovascular disease) [23] it is very well possible that these male smokers died of another disease before they could develop cancer. This explains the negative association between eosinophils and cancer mortality in the group with the highest risk for cardiovascular diseases (i.e., male smokers). Finally, a more mechanistic explanation for the negative association between eosinophils and cancer is that eosinophils release cytokines, which may lead to an antitumor response [24] and produce granule proteins that are highly cytotoxic for cancer cells [25]. It has also been proposed that a hyperactive immune function among smokers can detect and destroy malignant cells which may lead to an inverse association between allergies and cancer [10].

In our study, IgE was positively associated with lung cancer mortality among females. This finding may be explained by the fact that the lung is an organ which is directly exposed to the noxious stimuli which can be both allergens and carcinogens. This direct exposure induces excessive inflammation in allergic subjects which in turn may promote tumor development (i.e., the antigen stimulation theory may apply here) [14]. However, the gender difference has not been satisfactorily explained, but that is a common phenomenon in allergy research [26].

Higher levels of serum total IgE were associated with a decreased risk of hospitalization due to any cancer among smokers and males. As mentioned before, this can be a consequence of male smokers being more prone to develop lethal cardiovascular diseases. Another explanation may be that IgE antibodies physiologically survey tumor cells and eosinophils, and in addition, mast cells and macrophages can be armed with the cytophilic IgE. These all together become potent antitumor effectors, able to trace and kill tumor cells in the tissues [27]. However, evidence shows that an increased serum total IgE levels may not be exclusively related with atopic diseases [15].

Some other studies reported both inverse associations between allergy and cancer as well as positive associations [7], as we found. Therefore, our findings support the fact that the association between allergies and cancer is site specific. According to Sherman’s review, inverse associations were frequently reported for colorectal cancer, whereas a positive association was reported for lung cancer [7].

An important concern in the association between allergy and cancer is the potential effect modification of gender and smoking (3). A previous study assessing whether the association between history of asthma and/or hay fever and cancer mortality was modified by gender or smoking showed that the effect of asthma is more pronounced in males and ever smokers [28]. Although our results are in accordance with these findings, the possibility that these interaction results are confounded, by an unmeasured risk factor for allergy or cancer, is still of concern and warrants further studies.

Our study has several strengths. First, we investigated several common types of cancer (lung, colorectal, prostate, and breast cancer) whereas most other studies focused only on one specific type of cancer. Second, most previous studies were cross-sectional in design, whereas our cohort was followed up for over 40 years. Third, among previous studies, there is a failure to control for important cancer and allergy risk factors such as smoking and gender [1]. We studied three biological markers of allergic disease to operationalize allergy, whereas the majority of previous studies used questionnaires or investigated associations between cancer types and asthma, assuming an underlying atopic constitution that was not tested formally using objective allergy tests. Finally, a high follow-up rate should be mentioned, as 99.7 % of the included subjects could be traced back (Online Resource Table 8).

Hospitalization data were only available since 1995 and were obtained using probabilistic methods to identify true matches, which can be subject to error and could be considered a limitation of our study. Another limitation is that we studied both cancer mortality and hospital admissions as proxies for cancer incidence thereby assuming that the mechanisms relating allergy to cancer incidence do not differ from the mechanisms relating allergy to cancer mortality or hospitalization. Finally, no information was available on outpatient visits in the hospitalization data; therefore, we might have missed some cancer patients who did not require hospitalization.

In conclusion, our results indicate that we failed to identify overall associations between allergy markers and cancer. However, we found an inverse association between eosinophils, skin test positivity, and high serum total IgE and mortality and hospitalization from different types of cancer in specific subgroups. Hence, only studies on allergy and cancer that analyze sub-cohorts defined by gender and smoking habits may result in the possible identification of markers of predictive value.

## Electronic supplementary material

Below is the link to the electronic supplementary material.

Supplementary material 1 (TIFF 716 kb)

Supplementary material 2 (TIFF 715 kb)

Supplementary material 3 (TIFF 722 kb)

Supplementary material 4 (TIFF 705 kb)

Supplementary material 5 (DOCX 48 kb)

## Abbreviations

FEV_1_: Forced expiratory volume in 1 s
IgE: Immunoglobulin E
HR: Hazard ratio
OR: Odds ratio
ICD: International classification of diseases
BMI: Body mass index

## References

[1] Wang H, Diepgen TL (2005). Is atopy a protective or a risk factor for cancer? A review of epidemiological studies. Allergy.

[2] Engkilde K, Thyssen JP, Menne T, Johansen JD (2011). Association between cancer and contact allergy: a linkage study. BMJ Open.

[3] Turner MC, Chen Y, Krewski D, Ghadirian P (2006). An overview of the association between allergy and cancer. Int J Cancer.

[4] Merrill RM, Isakson RT, Beck RE (2007). The association between allergies and cancer: what is currently known?. Ann Allergy Asthma Immunol.

[5] Ji J, Shu X, Li X, Sundquist K, Sundquist J, Hemminki K (2009). Cancer risk in hospitalised asthma patients. Br J Cancer.

[6] Wang H, Rothenbacher D, Low M, Stegmaier C, Brenner H, Diepgen TL (2006). Atopic diseases, immunoglobulin E and risk of cancer of the prostate, breast, lung and colorectum. Int J Cancer.

[7] Sherman PW, Holland E, Sherman JS (2008). Allergies: their role in cancer prevention. Q Rev Biol.

[8] Josephs DH, Spicer JF, Corrigan CJ, Gould HJ, Karagiannis SN (2013). Epidemiological associations of allergy, IgE and cancer. Clin Exp Allergy.

[9] Olson SH, Hsu M, Satagopan JM, Maisonneuve P, Silverman DT, Lucenteforte E (2013). Allergies and risk of pancreatic cancer: a pooled analysis from the Pancreatic Cancer Case–Control Consortium. Am J Epidemiol.

[10] Hsiao JR, Ou CY, Lo HI, Huang CC, Lee WT, Huang JS (2013). Allergies and risk of head and neck cancer: an original study plus meta-analysis. PLoS One.

[11] Talbot-Smith A, Fritschi L, Divitini ML, Mallon DF, Knuiman MW (2003). Allergy, atopy, and cancer: a prospective study of the 1981 Busselton cohort. Am J Epidemiol.

[12] Hospers JJ, Schouten JP, Weiss ST, Postma DS, Rijcken B (2000). Eosinophilia is associated with increased all-cause mortality after a follow-up of 30 years in a general population sample. Epidemiology.

[13] van der Lende R, Kok TJ, Reig RP, Quanjer PH, Schouten JP, Orie NG (1981). Decreases in VC and FEV1 with time: indicators for effects of smoking and air pollution. Bull Eur Physiopathol Respir.

[14] Mensinga TT, Schouten JP, Rijcken B, Weiss ST, Speizer FE, Van der Lende R (1990). The relationship of eosinophilia and positive skin test reactivity to respiratory symptom prevalence in a community-based population study. J Allergy Clin Immunol.

[15] Jansen DF, Rijcken B, Schouten JP, Kraan J, Weiss ST, Timens W, Postma DS (1999). The relationship of skin test positivity, high serum total IgE levels, and peripheral blood eosinophilia to symptomatic and asymptomatic airway hyperresponsiveness. J AM Respir Med.

[16] Prentice RL, Kalbfleisch JD, Peterson AV, Flournoy N, Farewell VT, Breslow NE (1978). The analysis of failure times in the presence of competing risks. Biom J.

[17] Rittmeyer D, Lorentz A (2012). Relationship between allergy and cancer: an overview. Int Arch Allergy Immunol.

[18] Turner MC (2012). Epidemiology: allergy history, IgE, and cancer. Cancer Immunol Immunother.

[19] Vojtechova P, Martin RM (2009). The association of atopic diseases with breast, prostate, and colorectal cancers: a meta-analysis. Cancer Causes Control.

[20] Eriksson NE, Mikoczy Z, Hagmar L (2005). Cancer incidence in 13811 patients skin tested for allergy. J Investig Allergol Clin Immunol.

[21] Lowcock EC, Cotterchio M, Ahmad N (2013). Association between allergies, asthma, and breast cancer risk among women in Ontario, Canada. Cancer Causes Control.

[22] Mensinga TT (1993) Smoking modifies the relationship of markers of allergy to eosinophil count in a community-based population study. In: Thesis, University of Groningen, Groningen, The Netherlands vol 3, pp 39–58

[23] Tanaka T, Ozaki K (2006). Inflammation as a risk factor for myocardial infarction. J Hum Genet.

[24] Legrand F, Driss V, Delbeke M, Loiseau S, Hermann E, Dombrowicz D (2010). Human eosinophils exert TNF-α and granzyme A-mediated tumoricidal activity toward colon carcinoma cells. J Immunol.

[25] Munitz A, Levi-Schaffer F (2004). Eosinophils: ‘new’ roles for ‘old’ cells. Allergy.

[26] Chen W, Mempel M, Schober W, Behrendt H, Ring J (2008). Gender difference, sex hormones, and immediate type hypersensitivity reactions. Allergy.

[27] Jensen-Jarolim E, Achatz G, Turner MC, Karagiannis S, Legrand F, Capron M (2008). AllergoOncology: the role of IgE-mediated allergy in cancer. Allergy.

[28] Turner MC, Chen Y, Krewski D, Ghadirian P, Thun MJ, Calle EE (2005). Cancer mortality among US men and women with asthma and hay fever. Am J Epidemiol.

