# Supplementary figures and images for: Objective allergy markers and risk of cancer mortality and hospitalization in a large population-based cohort
Source: Cancer Causes Control. 2014 Nov 12;26(1):99–109. doi: 10.1007/s10552-014-0489-9 (PMC4282688; doi:10.1007/s10552-014-0489-9)

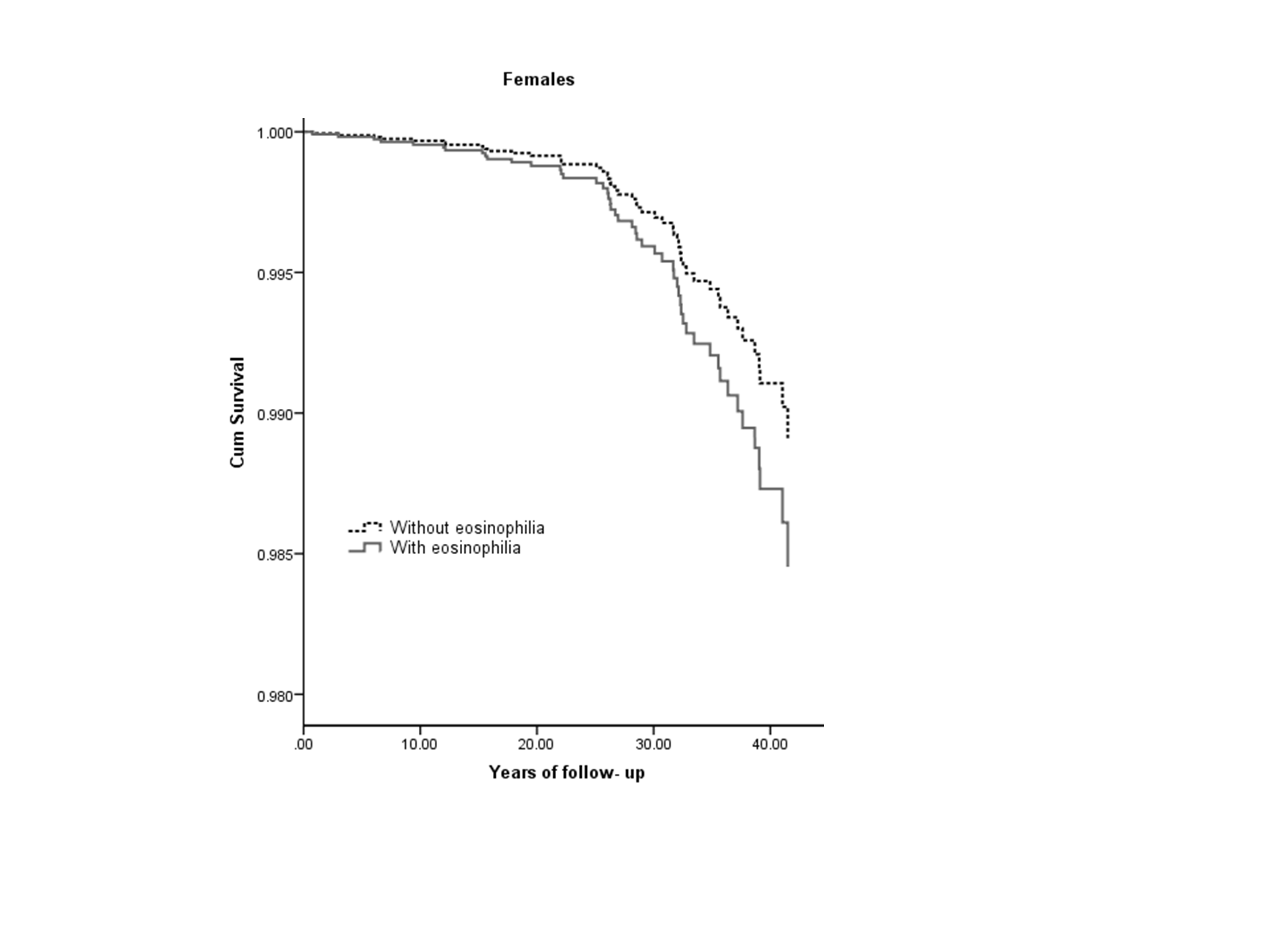

Supplement: Supplementary file 1 — Supplementary material 1 (TIFF 716 kb) [file 10552_2014_489_MOESM1_ESM.tif]

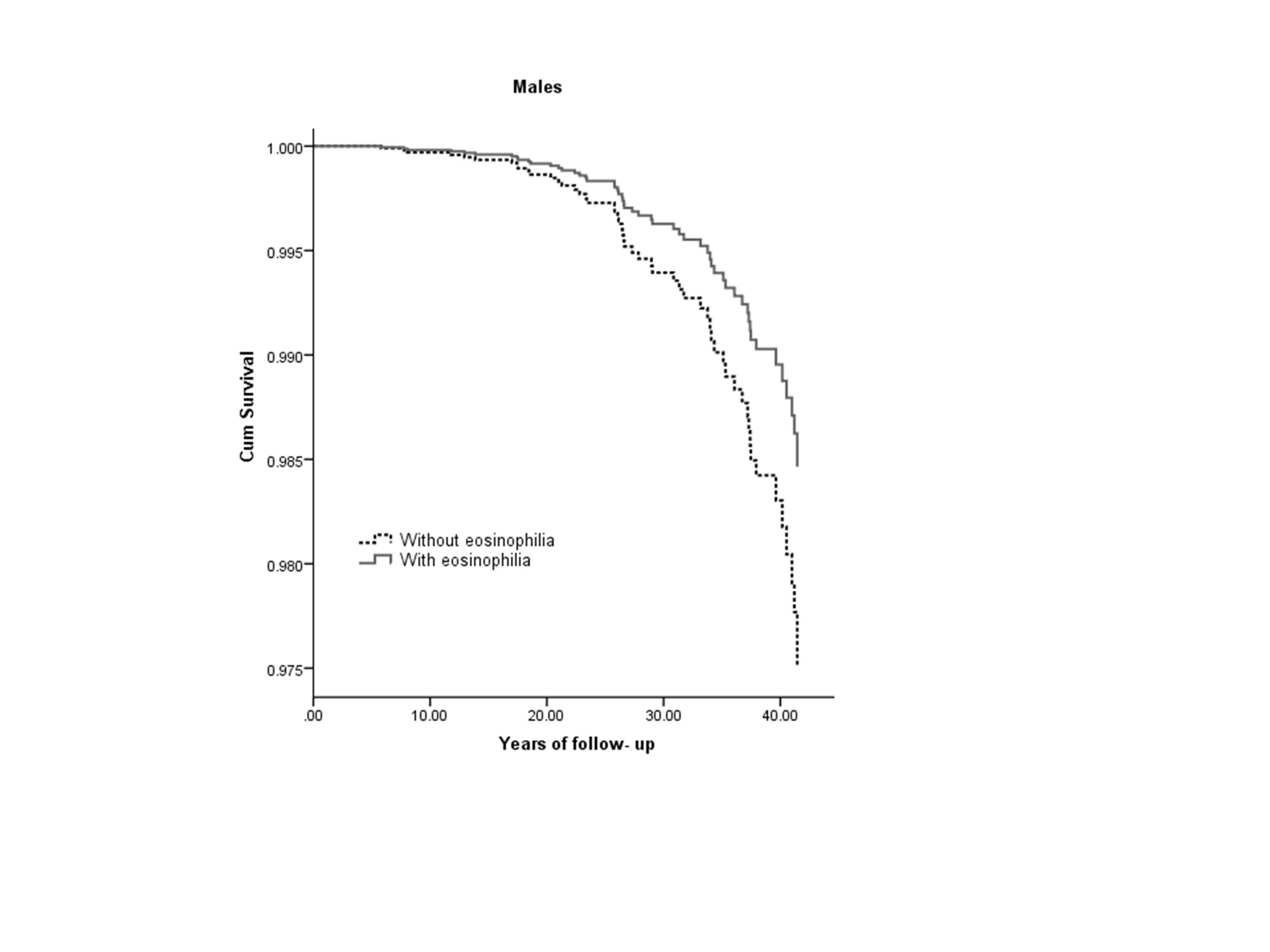

Supplement: Supplementary file 2 — Supplementary material 2 (TIFF 715 kb) [file 10552_2014_489_MOESM2_ESM.tif]

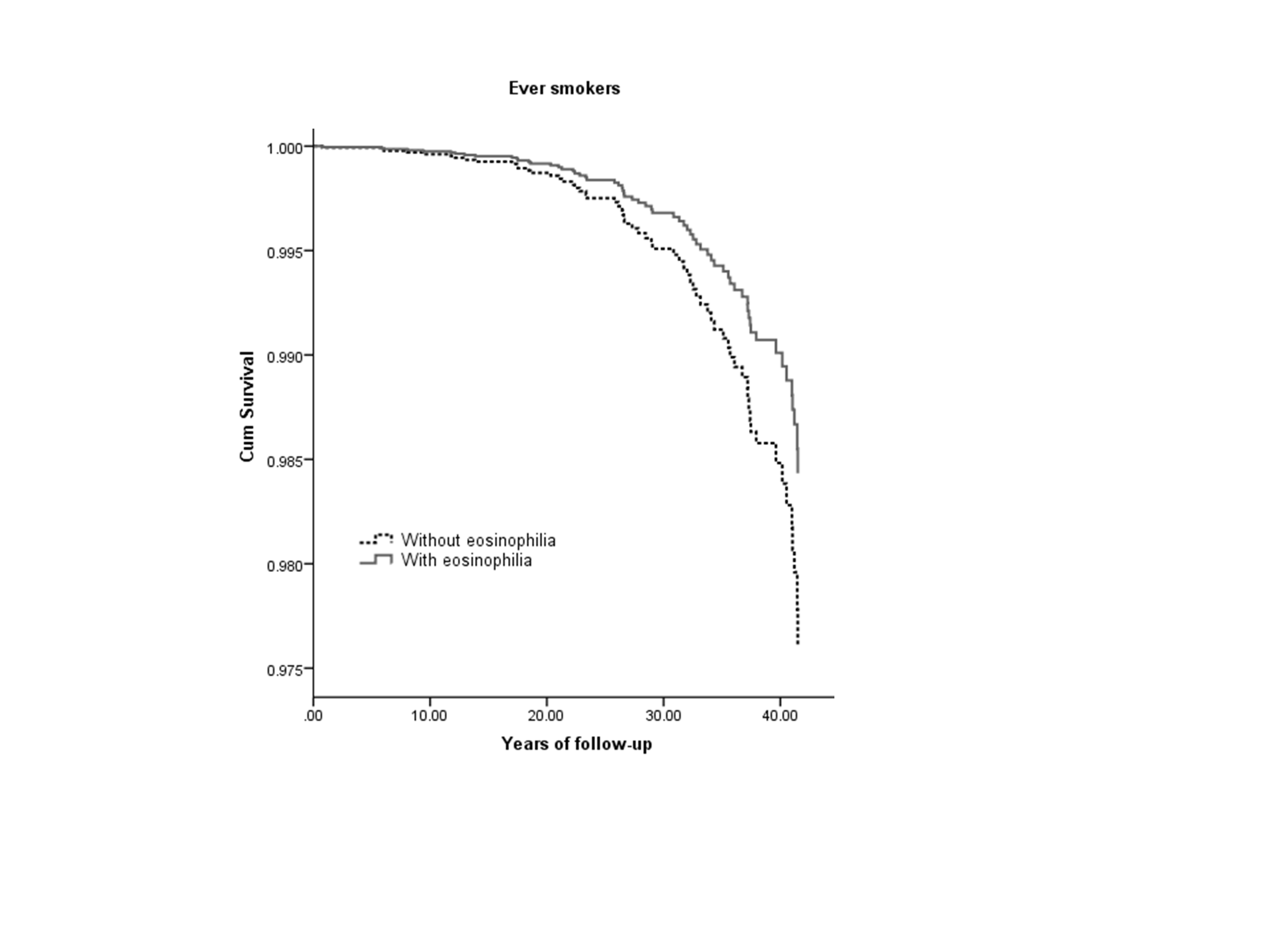

Supplement: Supplementary file 3 — Supplementary material 3 (TIFF 722 kb) [file 10552_2014_489_MOESM3_ESM.tif]

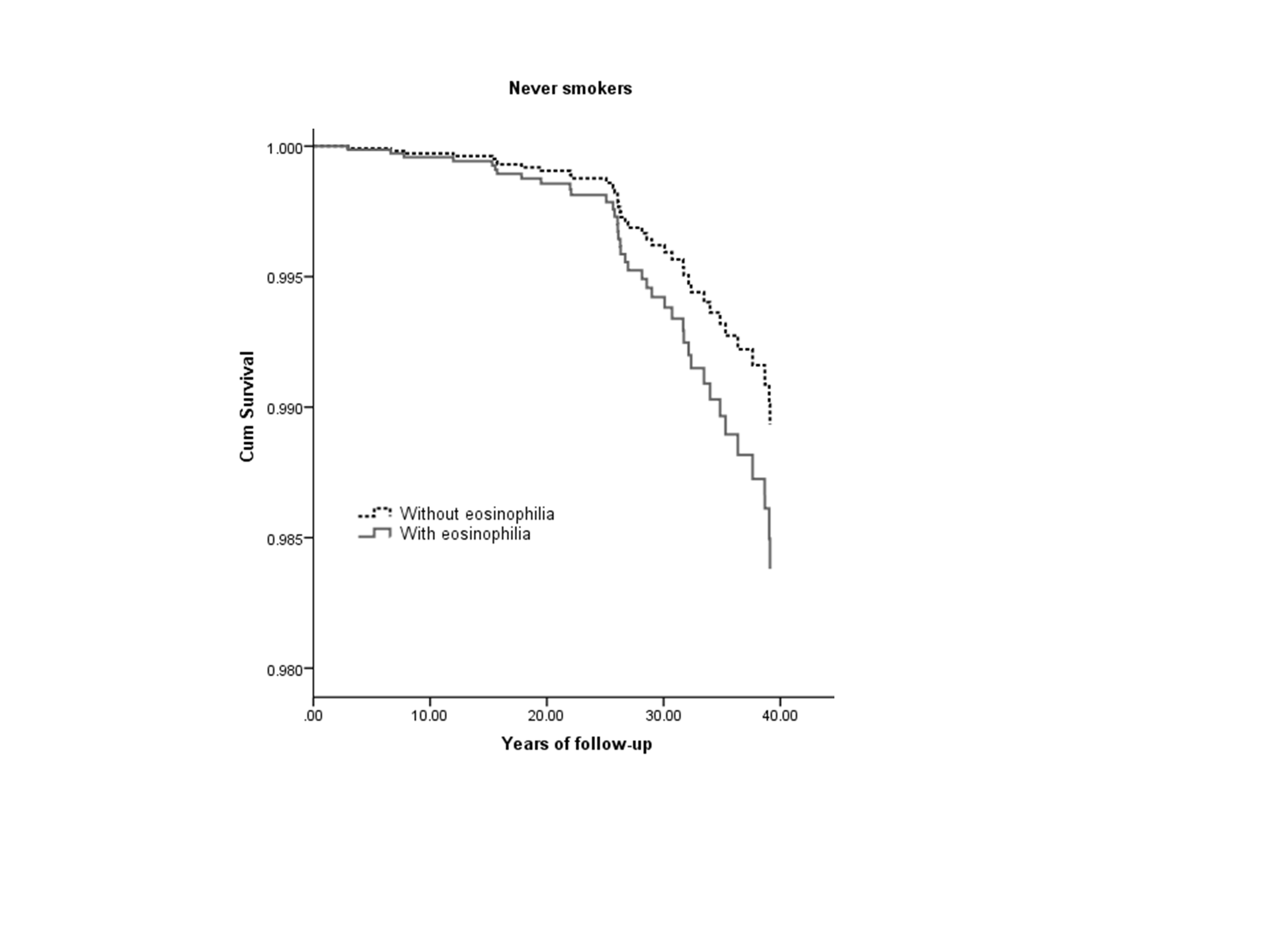

Supplement: Supplementary file 4 — Supplementary material 4 (TIFF 705 kb) [file 10552_2014_489_MOESM4_ESM.tif]
