# Supplementary material for: Objective allergy markers and risk of cancer mortality and hospitalization in a large population-based cohort
Source: Cancer Causes Control. 2014 Nov 12;26(1):99–109. doi: 10.1007/s10552-014-0489-9 (PMC4282688; doi:10.1007/s10552-014-0489-9)
Supplement: Supplementary file 5 — Supplementary material 5 (DOCX 48 kb) [file 10552_2014_489_MOESM5_ESM.docx]

***Cancer Causes & Control (CCC)***

**Objective allergy markers and risk of cancer mortality and hospitalization in a large population-based cohort**

Niloofar Taghizadeh^1^, MSc, Judith M. Vonk^1,2^, MD. PhD, Jeannette J. Hospers^1,2^, PhD, Dirkje S. Postma^2,3,^, PhD, Liesbeth de Vries^4^, MD. PhD, Jan P. Schouten^1^, PhD, H. Marike Boezen^1,2^, PhD

1. University of Groningen, University Medical Center Groningen, Department of Epidemiology

2. University of Groningen, University Medical Center Groningen, GRIAC Research Institute

3. University of Groningen, University Medical Center Groningen, Department of Pulmonology

4. University of Groningen, University Medical Center Groningen, Department of Medical Oncology

Corresponding author:

HM Boezen, PhD

Department of Epidemiology

University Medical Center Groningen

Hanzeplein 1

9700 RD Groningen

The Netherlands

Telephone: +31 (0) 50 361 0739

Fax: + 31 (0) 50 361 4493

Email: [H.M.Boezen@umcg.nl](mailto:H.M.Boezen@umcg.nl)

**Online Resource Table 1-** Characteristics at the first survey of subjects without (EO-) and with eosinophilia (EO+) (eosinophilia defined as > 275 cells/mm^3^), and without (ST-) and with positive skin tests (ST+).

|  | **EO-** | **EO+** | **p-value ^b^** | **ST-** | **ST+** | **p-value ^b^** |
| --- | --- | --- | --- | --- | --- | --- |
|  |  |  |  |  |  |  |
| All subjects, n (%) | 6416 (88.1) | 868 (11.9) |  | 5402 (85.0) | 957 (15.0) |  |
| Men, % | 51.1 | 55.2 | 0.03 | 50.7 | 59.2 | 0.00 |
| Age (years), mean (SD) | 36.6 (13.4) | 36.4 (13.9) | 0.70 | 37.2 (13.7) | 30.8 (11.6) | 0.00 |
| Smoking , % |  |  |  |  |  |  |
| Never smoker | 36.5 | 32.8 | 0.20 | 38.8 | 33.1 | 0.00 |
| Ever smoker | 63.5 | 67.2 |  | 61.2 | 66.9 |  |
| FEV_1_% predicted ^a^, mean (SD) | 88.3 (13.9) | 84.4 (15.7) | 0.00 | 87.2 (14.3) | 87.8 (13.9) | 0.17 |
| BMI (kg/m^2^), % |  |  |  |  |  |  |
| <25 | 51.1 | 54.1 | 0.05 | 49.4 | 60.6 |  |
| 25-30 | 38.1 | 33.9 |  | 39.0 | 32.6 | 0.00 |
| >30 | 10.7 | 12.0 |  | 11.6 | 6.8 |  |
| Place of residence, % |  |  |  |  |  |  |
| Vlagtwedde | 63.9 | 49.8 | 0.00 | 58.8 | 44.6 | 0.00 |
|  |  |  |  |  |  |  |

^a^ FEV_1_% of predicted, percentage of predicted forced expiratory volume in 1 second.

^b^ P-value calculated by Chi- square or t-test.

**Online Resource Table 2-** Vital status in 2008 for subjects without (EO-) and with eosinophilia (EO+) (eosinophilia defined as > 275 cells/mm^3^), and without (ST-) and with positive skin tests (ST+) at the first survey.

|  | **EO-** | **EO+** | **p-value** | **ST-** | **ST+** | **p-value** |
| --- | --- | --- | --- | --- | --- | --- |
|  |  |  |  |  |  |  |
| Alive | 59.9 | 55.1 | 0.01^a^ | 53.2 | 69.7 | 0.00^a^ |
| Died due to cancer | 13.2 | 12.1 |  | 15.2 | 9.4 |  |
| Died but not due to cancer | 23.8 | 28.3 |  | 28.8 | 16.9 |  |
| Died due to external causes | 1.6 | 2.2 |  | 1.8 | 1.6 |  |
| Lost to follow-up | 1.5 | 2.3 |  | 1.1 | 2.4 |  |
| **Mortality due to specific types of cancer** |  |  |  |  |  |  |
| Lung cancer | 24.2 | 22.9 | 0.76^b^ | 22.4 | 25.6 | 0.49^b^ |
| Colorectal cancer | 11.4 | 10.5 | 0.78 | 11.2 | 13.3 | 0.56 |
| Prostate cancer | 10.8 | 8.0 | 0.51 | 10.1 | 4.0 | 0.43 |
| Breast cancer | 11.8 | 4.0 | 0.02 | 10.3 | 6.2 | 0.20 |
|  |  |  |  |  |  |  |

^a^ P- value calculated by Chi- square within vital status (alive, died due to cancer, not due to cancer and died due to external causes)

^b^ P- value calculated by Chi- square compare to died due to other cancer types.

| **All subjects** | **Any cancer** | **Lung cancer** | **Colorectal cancer** | **Prostate cancer** | **Breast cancer** |
| --- | --- | --- | --- | --- | --- |
|  |  |  |  |  |  |
| **Cancer mortality** | **events/censored** | **events/censored** | **events/censored** | **events/censored** | **events/censored** |
|  | n (%)/n (%) | n (%)/n (%) | n (%)/n (%) | n (%)/n (%) | n (%)/n (%) |
| Eosinophils (ln) | 921 (13.0)/6164 (87.0) | 223 (3.1)/6862 (96.9) | 102 (1.4)/6983 (98.6) |  |  |
| Skin test positivity | 875 (12.4)/5318 (87.6) | 200 (3.2)/5993 (96.8) | 97 (1.5)/6096 (98.5) |  |  |
| Total IgE (log10) | 212 (9.1)/2112 (90.9) | 43 (1.8)/2281 (98.2) | 27 (1.2)/2297 (98.8) |  |  |
|  |  |  |  |  |  |
| **Cancer hospitalization** | **hosp/no hosp** | **hosp/no hosp** | **hosp/no hosp** | **hosp/no hosp** | **hosp/no hosp** |
| Eosinophils (ln) | 914 (16.8)/4535 (83.2) | 103 (1.9)/5346 (98.1) | 95 (1.7)/5354 (98.3) |  |  |
| Skin test positivity | 798 (17.3)/3813 (82.7) | 94 (2.0)/4517 (98.0) | 89 (3.9)/4522 (98.1) |  |  |
| Total IgE (log10) | 384 (16.7)/1915 (83.3) | 39 (1.7)/2260 (98.3) | 50 (2.2)/2249 (97.8) |  |  |
|  |  |  |  |  |  |

**Online Resource Table 3-** Number of subjects included in the analyses on the associations between allergy markers and mortality and hospitalization from any and specific type of cancer.

| **Females** | **Any cancer** | **Lung cancer** | **Colorectal cancer** | **Prostate cancer** | **Breast cancer** |
| --- | --- | --- | --- | --- | --- |
|  |  |  |  |  |  |
| **Cancer mortality** | **events/censored** | **events/censored** | **events/censored** | **events/censored** | **events/censored** |
|  | n (%)/n (%) | n (%)/n (%) | n (%)/n (%) | n (%)/n (%) | n (%)/n (%) |
| Eosinophils (ln) | 372 (10.9)/3035 (89.1) | 40 (1.2)/3367 (98.8) | 49 (1.4)/3358 (98.6) |  | 92 (2.7)/3315 (97.3) |
| Skin test positivity | 354 (12.0)/2600 (88.0) | 32 (1.1)/2922 (98.9) | 46 (1.6)/2908 (98.4) |  | 90 (3.0)/2864 (97.0) |
| Total IgE (log10) | 63 (5.9)/1006 (94.1) | 5 (0.5)/1064 (99.5) | 9 (0.8)/1060 (99.2) |  | 10 (0.9)/1059 (99.1) |
|  |  |  |  |  |  |
| **Cancer hospitalization** | **hosp/no hosp** | **hosp/no hosp** | **hosp/no hosp** | **hosp/no hosp** | **hosp/no hosp** |
| Eosinophils (ln) | 445 (16.2)/2308 (83.8) | 20 (0.7)/2733 (99.3) | 42 (1.5)/2711 (98.5) |  | 85 (3.1)/2668 (96.9) |
| Skin test positivity | 382 (16.4)/1947 (83.6) | 19 (0.8)/2310 (99.2) | 40 (1.7)/2289 (98.3) |  | 74 (3.2)/2255 (96.8) |
| Total IgE (log10) | 164 (15.2)/915 (84.8) | 5 (0.5)/1074 (99.5) | 17 (1.6)/1062 (98.4) |  | 30 (2.8)/1049 (97.2) |
|  |  |  |  |  |  |

| **Males** | **Any cancer** | **Lung cancer** | **Colorectal cancer** | **Prostate cancer** | **Breast cancer** |
| --- | --- | --- | --- | --- | --- |
|  |  |  |  |  |  |
| **Cancer mortality** | **events/censored** | **events/censored** | **events/censored** | **events/censored** | **events/censored** |
|  | n (%)/n (%) | n (%)/n (%) | n (%)/n (%) | n (%)/n (%) | n (%)/n (%) |
| Eosinophils (ln) | 549 (14.9)/3129 (85.1) 57.45757(((57.4 | 183 (5.0)/3495 (95.0) | 53 (1.4)/3625 (98.6) | 59 (1.6)/3619 (98.4) |  |
| Skin test positivity | 521 (16.1)/2718 (83.9) | 168 (5.2)/3071 (94.8) | 51 (1.6)/3188 (98.4) | 58 (1.8)/3181 (98.2) |  |
| Total IgE (log10) | 149 (11.9)/1106 (88.1) | 38 (3.0)/1217 (97.0) | 18 (1.4)/1237 (98.6) | 17 (1.4)/1238 (98.6) |  |
|  |  |  |  |  |  |
| **Cancer hospitalization** | **hosp/no hosp** | **hosp/no hosp** | **hosp/no hosp** | **hosp/no hosp** | **hosp/no hosp** |
| Eosinophils (ln) | 469 (17.4)/2227 (82.6) | 83 (3.1)/2613 (96.9) | 53 (2.0)/2643 (98.0) | 84 (3.1)/2612 (96.9) |  |
| Skin test positivity | 416 (18.2)/1866 (81.8) | 75 (3.3)/2207 (96.7) | 49 (2.1)/2233 (97.9) | 75 (3.3)/2207 (96.7) |  |
| Total IgE (log10) | 220 (8.0)/1000 (82.0) | 34 (2.8)/1186 (97.2) | 33 (2.7)/1187 (97.3) | 43 (3.5)/1177 (96.5) |  |
|  |  |  |  |  |  |

| **Ever smokers** | **Any cancer** | **Lung cancer** | **Colorectal cancer** | **Prostate cancer** | **Breast cancer** |
| --- | --- | --- | --- | --- | --- |
|  |  |  |  |  |  |
| **Cancer mortality** | **events/censored** | **events/censored** | **events/censored** | **events/censored** | **events/censored** |
|  | n (%)/n (%) | n (%)/n (%) | n (%)/n (%) | n (%)/n (%) | n (%)/n (%) |
| Eosinophils (ln) | 651 (14.3)/3897 (85.7) | 213 (4.7) /4335 (95.3) (61.2) | 63 (1.4)/4485 (98.6) | 52 (1.7)/3050 (98.3) | 30 (2.0)/1444 (98.0) |
| Skin test positivity | 607 (15.7)/3259 (84.3) | 191 (4.9)/3675 (95.1) | 59 (1.5)/3807 (98.5) | 51 (1.9)/2678 (98.1) | 27 (2.4)/1110 (97.6) |
| Total IgE (log10) | 169 (10.9)/1378 (89.1) | 42 (2.7)/1505 (97.3) | 22 (1.4)/1525 (98.6) | 16 (1.5)/1075 (98.5) | 3 (0.6)/512 (99.4) |
|  |  |  |  |  |  |
| **Cancer hospitalization** | **hosp/no hosp** | **hosp/no hosp** | **hosp/no hosp** | **hosp/no hosp** | **hosp/no hosp** |
| Eosinophils (ln) | 628 (18.2)/2823 (81.8) | 97 (2.8)/3354 (97.2) | 66 (1.9)/3385 (98.1) | 75 (3.4)/2116 (96.6) | 33 (2.6)/1227 (97.4) |
| Skin test positivity | 541 (19.2)/2275 (80.8) | 89 (3.2)/2727 (96.8) | 59 (2.1)/2757 (97.9) | 68 (3.7)/1770 (96.3) | 26 (2.7)/952 (97.3) |
| Total IgE (log10) | 280 (18.6)/1228 (81.4) | 37 (2.5)/1473 (97.5) | 39 (2.6)/1471 (97.4) | 38 (3.7)/981 (96.3) | 13 (2.6)/478 (97.4) |
|  |  |  |  |  |  |

| **Never smokers** | **Any cancer** | **Lung cancer** | **Colorectal cancer** | **Prostate cancer** | **Breast cancer** |
| --- | --- | --- | --- | --- | --- |
|  |  |  |  |  |  |
| **Cancer mortality, n (%)** | **events/censored** | **events/censored** | **events/censored** | **events/censored** | **events/censored** |
|  | n (%)/n (%) | n (%)/n (%) | n (%)/n (%) | n (%)/n (%) | n (%)/n (%) |
| Eosinophils (ln) | 270 (10.6)/2267 (89.4) | 10 (0.4)/2527 (99.6) | 39 (1.5)/2498 (98.5) | 7 (1.2)/567 (98.8) | 62 (3.2)/1901 (96.8) |
| Skin test positivity | 268 (13.4)/2059 (86.6) | 9 (0.4)/2318 (99.6) | 38 (1.6)/2289 (98.4) | 7 (1.4)/503 (98.6) | 63 (3.5)/1754 (96.5) |
| Total IgE (log10) | 42 (5.4)/729 (94.6) | 1 (0.1)/770 (99.9) | 5 (0.6)/766 (99.4) | 1 (0.5)/204 (99.5) | 8 (1.3)/606 (98.7) |
|  |  |  |  |  |  |
| **Cancer hospitalization, n (%)** | **hosp/no hosp** | **hosp/no hosp** | **hosp/no hosp** | **hosp/no hosp** | **hosp/no hosp** |
| Eosinophils (ln) | 286 (14.3)/1712 (85.7) | 6 (0.3)/1992 (99.7) | 29 (1.5)/1969 (98.5) | 9 (1.8)/496 (98.2) | 52 (3.5)/1441 (96.5) |
| Skin test positivity | 257 (14.3)/1538 (85.7) | 5 (0.2)/1790 (99.8) | 30 (1.7)/1765 (98.3) | 7 (1.6)/437 (98.4) | 48 (1.0)/1303 (96.4) |
| Total IgE (log10) | 103 (13.1)/686 (86.9) | 2 (0.3)/787 (99.7) | 11 (1.4)/778 (98.6) | 5 (2.5)/196 (97.5) | 17 (2.9)/571 (97.1) |
|  |  |  |  |  |  |

**Online Resource Table 4-** Interaction of eosinophils with smoking habits stratified by gender and interaction of eosinophils with gender stratified by smoking habits on colorectal cancer mortality risk.

| **Colorectal cancer mortality HR ( 95% CI)** | | | | | |
| --- | --- | --- | --- | --- | --- |
|  |  | **P-value** |  |  | **P-value** |
|  | Male |  |  | Female |  |
| Effect in never smokers | 1.44 (0.42-4.96) | 0.563 |  | 1.50 (0.91-2.48) | 0.114 |
| Effect in ever smokers | **0.57 (0.40-0.81)** | 0.002 |  | 0.84 (0.45-1.57) | 0.587 |
| Interaction | 0.40 (0.11-1.43) | 0.157 |  | 0.56 (0.25-1.25) | 0.157 |
|  |  |  |  |  |  |
|  | Ever smoker |  |  | Never smoker |  |
| Effect in females | 0.85 (0.45-1.61) | 0.612 |  | 1.47 (0.90-2.41) | 0.127 |
| Effect in males | **0.56 (0.39-0.80)** | 0.001 |  | 1.35 (0.39-4.67) | 0.634 |
| Interaction | 0.66 (0.32-1.36) | 0.261 |  | 0.92 (0.24-3.49) | 0.902 |
|  |  | | |  | |

Statistically significant results are shown in bold.

**Online Resource Table 5-** Interaction of eosinophils, skin test positivity and IgE **with smoking** on cancer hospitalization risk.

|  | **Any cancer**  **OR ( 95% CI)** | **P-value** | **Lung cancer**  **OR ( 95% CI)** | **P-value** | **Colorectal cancer**  **OR ( 95% CI)** | **P-value** | **Prostate cancer**  **OR ( 95% CI)** | **P-value** | **Breast cancer**  **OR ( 95% CI)** | **P-value** |
| --- | --- | --- | --- | --- | --- | --- | --- | --- | --- | --- |
|  |  |  |  |  |  |  |  |  |  |  |
| Eosinophils (ln) |  |  |  |  |  |  |  |  |  |  |
| Effect in never smokers | 1.04 (0.88-1.22) | 0.875 | 1.31 (0.44-3.86) | 0.872 | 1.49 (0.90-2.47) | 0.682 | 1.11 (0.46-2.70) | 0.936 | 0.94 (0.66-1.34) | 0.886 |
| Effect in ever smokers | 0.96 (0.85-1.08) | 0.743 | 1.16 (0.87-1.55) | 0.767 | 0.90 (0.65-1.25) | 0.765 | 0.97 (0.70-1.34) | 0.942 | 1.06 (0.68-1.64) | 0.928 |
| Interaction | 0.92 (0.76-1.13) | 0.678 | 0.89 (0.29-2.73) | 0.936 | 0.60 (0.33-1.09) | 0.167 | 0.87 (0.34-2.23) | 0.905 | 1.13 (0.64-1.98) | 0.887 |
|  |  |  |  |  |  |  |  |  |  |  |
| Skin test positivity |  |  |  |  |  |  |  |  |  |  |
| Effect in never smokers | 0.97 (0.66-1.43) | 0.952 | 0.00 (0.00-∞) | NA | 1.35 (0.51-3.61) | 0.847 | 0.73 (0.09-6.19) | 0.905 | 0.92 (0.38-2.21) | 0.942 |
| Effect in ever smokers | 0.98 (0.76-1.26) | 0.951 | 0.78 (0.42-1.46) | 0.692 | 0.57 (0.24-1.35) | 0.405 | 1.25 (0.67-2.35) | 0.827 | 1.19 (0.44-3.23) | 0.908 |
| Interaction | 1.01(0.64-1.60) | 0.988 | 3590841 (0.00-∞) | NA | 0.42 (0.12-1.55) | 0.382 | 1.71 (0.19-15.79) | 0.876 | 1.30 (0.35-4.85) | 0.896 |
|  |  |  |  |  |  |  |  |  |  |  |
| Total IgE (log10) |  |  |  |  |  |  |  |  |  |  |
| Effect in never smokers | 1.04 (0.73-1.48) | 0.940 | 1.61 (0.16-15.94) | 0.892 | 0.41 (0.13-1.27) | 0.243 | 0.38 (0.07-1.97) | 0.485 | 1.13 (0.51-2.51) | 0.918 |
| Effect in ever smokers | 0.80 (0.64-1.00) | 0.050 | 0.86 (0.49-1.51) | 0.812 | 1.13 (0.66-1.94) | 0.883 | 0.90 (0.52-1.56) | 0.873 | 0.63 (0.24-1.65) | 0.605 |
| Interaction | 0.77 (0.51-1.17) | 0.435 | 0.54 (0.05-5.70) | 0.817 | 2.76 (0.79-9.66) | 0.676 | 2.36 (0.42-13.35) | 0.773 | 0.56 (0.16-1.94) | 0.620 |
|  |  |  |  |  |  |  |  |  |  |  |

NA=Not Available, no mortality in this category.

**Online Resource 6-** Interaction of eosinophils, skin test positivity and IgE **with gender** on cancer hospitalization risk.

|  | **Any cancer**  **OR ( 95% CI)** | **P-value** | **Lung cancer**  **OR ( 95% CI)** | **P-value** | **Colorectal cancer**  **OR ( 95% CI)** | **P-value** |
| --- | --- | --- | --- | --- | --- | --- |
|  |  |  |  |  |  |  |
| Eosinophils (ln) |  |  |  |  |  |  |
| Effect in females | 0.97 (0.85-1.11) | 0.845 | 1.39 (0.78-2.49) | 0.749 | 1.28 (0.85-1.92) | 0.735 |
| Effect in males | 1.00 (0.87-1.15) | 1.000 | 1.11 (0.81-1.52) | 0.836 | 0.88 (0.61-1.28) | 0.744 |
| Interaction | 1.03 (0.85-1.25) | 0.918 | 0.80 (0.41-1.54) | 0.748 | 0.69 (0.40-1.20) | 0.379 |
|  |  |  |  |  |  |  |
| Skin test positivity |  |  |  |  |  |  |
| Effect in females | 1.02 (0.75-1.41) | 0.965 | 0.70 (0.16-3.06) | 0.833 | 0.99 (0.38-2.57) | 0.994 |
| Effect in males | 0.94 (0.71-1.25) | 0.852 | 0.75 (0.38-1.49) | 0.669 | 0.65 (0.28-1.57) | 0.591 |
| Interaction | 0.92 (0.60-1.41) | 0.870 | 1.07 (0.21-5.45) | 0.977 | 0.66 (0.18-2.39) | 0.764 |
|  |  |  |  |  |  |  |
| Total IgE (log10) |  |  |  |  |  |  |
| Effect in females | 1.01 (0.76-1.35) | 0.980 | 2.70 (0.60-12.11) | 0.718 | 0.47 (0.19-1.15) | 0.181 |
| Effect in males | **0.76 (0.59-0.98)** | 0.016 | 0.75 (0.42-1.36) | 0.599 | 1.25 (0.70-2.24) | 0.815 |
| Interaction | 0.75 (0.51-1.10) | 0.285 | 0.28 (0.06-1.40) | 0.229 | 2.65 (0.91-7.71) | 0.650 |
|  |  |  |  |  |  |  |

Statistically significant result is shown in bold.

**Online Resource Table 7-** The hazard ratios of eosinophil levels in equally spaced categories for mortality from colorectal cancer in Vlagtwedde-Vlaardingen.

| **Colorectal cancer**  **HR ( 95% CI)** | **All subjects** | **Females** | **Males** | **Never smokers** | **Ever smokers** |
| --- | --- | --- | --- | --- | --- |
|  |  |  |  |  |  |
| Eosinophils (ln) |  |  |  |  |  |
| <1.75 | 1 | 1 | 1 | 1 | 1 |
| 1.75-2.15 | 0.78 (0.42-1.44) | 1.30 (0.49-3.44) | 0.54 (0.24-1.22) | 1.93 (0.59-6.31) | 0.51 (0.24-1.09) |
| 2.15-2.55 | 0.72 (0.40-1.31) | 1.03 (0.39-2.71) | 0.54 (0.25-1.17) | 1.20 (0.35-4.12) | 0.60 (0.30-1.19) |
| 2.55-2.95 | **0.46 (0.24-0.89)** | 0.81 (0.29-2.25) | **0.30 (0.13-0.73)** | 1.37 (0.40-4.71) | **0.28 (0.12-0.63)** |
| >2.95 | 0.72 (0.40-1.33) | 1.68 (0.67-4.19) | **0.33 (0.14-0.79)** | 2.31 (0.74-7.27) | **0.36 (0.17-0.80)** |

**Online Resource Table 8-** The number of subjects recruited in Vlagtwedde and Vlaardingen per year and the percent successfully followed up to December 31 2008.

|  | | **Total N** | | **Eosinophils** | | **Skin test positivity** | | **IgE** | **Vital status in 2008** | | | | |
| --- | --- | --- | --- | --- | --- | --- | --- | --- | --- | --- | --- | --- | --- |
|  | |  | |  | |  | |  | **Alive** | **Died due to cancer** | **Died, but not due to cancer** | **Died due to external** | **Lost to follow-up** |
| **Vlagtwedde** | |  | |  | |  | |  |  |  |  |  |  |
|  | | N | | N (%) | | N (%) | | N (%) | N (%) | N (%) | N (%) | N (%) | N (%) |
| 1965 | | 2151^a^ | | 1049 (48.8) | | 1194 (55.5) | | 161 (7.5) | 254 (11.8) | 466 (21.7) | 1345 (62.5) | 67 (3.1) | 13 (0.6) |
| 1967 | | 1793^b^ | | 1782 (99.4) | | 1784 (99.5) | | 708 (39.5) | 1445 (80.6) | 147 (8.2) | 160 (8.9) | 23 (1.3) | 16 (0.9) |
| 1970 | | 632 | | 624 (98.7) | | 625 (98.9) | | 267 (42.2) | 448 (70.9) | 71 (11.2) | 90 (14.2) | 13 (2.1) | 10 (1.6) |
| 1973 | | 297 | | 291 (98.0) | |  | | 99 (33.3) | 242 (81.5) | 15 (5.1) | 33 (11.1) | 4 (1.3) | 3 (1.0) |
| 1976 | | 200 | | 197 (98.5) | |  | | 74 (37.0) | 161 (80.5) | 16 (8.0) | 21 (10.5) | 1 (0.5) | 1 (0.5) |
| 1979 | | 165 | | 161 (97.6) | |  | | 72 (43.6) | 130 (78.8) | 8 (4.8) | 19 (11.5) | 1 (0.6) | 7 (4.2) |
| 1982 | | 129 | | 128 (99.2) | |  | | 60 (46.5) | 99 (76.7) | 8 (6.2) | 16 (12.4) | 1 (0.8) | 5 (3.9) |
| 1985 | | 123 | | 122 (99.2) | |  | | 42 (34.1) | 91 (74.0) | 5 (4.1) | 20 (16.3) | 2 (1.6) | 5 (4.1) |
| 1989 | | 185 | | 181 (97.8) | |  | | 176 (95.3) | 134 (72.4) | 26 (14.1) | 14 (7.6) | 3 (1.6) | 8 (4.3) |
| Total N | | 5675 | | 4535 | | 3603 | | 1659 | 3004 | 763 | 1718 | 115 | 68 |
| **Vlaardingen** | | | | | | | | | | | | | |
| 1965 | 1199 ^c^ | | 1181 (98.5) | | 1184 (98.4) | | 264 (22.0) | | 204 (17.1) | 313 (26.2) | 646 (54.0) | 25 (2.1) | 8 (0.7) |
| 1969 | 1591^d^ | | 1568 (98.6) | | 1571 (98.8) | | 524 (32.9) | | 1297 (81.7) | 118 (7.4) | 109 (6.9) | 18 (1.1) | 46 (2.9) |
| Total N | 2790 | | 2749 | | 2755 | | 788 | | 1501 | 431 | 755 | 43 | 54 |
|  |  | |  | |  | |  | |  |  |  |  |  |

^a^ In 6 subjects the cause of death could not be obtained.

^b^ In 2 subjects the cause of death could not be obtained.

^c^ In 3 subjects the cause of death could not be obtained.

^d^ In 2 subjects the cause of death could not be obtained.

|  |
| --- |
